# Determination of Redox Status in Different Tissues of Lambs and Kids and Their in-between Relationship

**DOI:** 10.3390/antiox11102065

**Published:** 2022-10-20

**Authors:** Zoi Skaperda, Ioannis D. Kyriazis, Fotios Tekos, Maria V. Alvanou, Paraskevi-Maria Nechalioti, Sotiria Makri, Angeliki Argyriadou, Sotiria Vouraki, Theodoros Kallitsis, Maria Kourti, Valasi Irene, Georgios Arsenos, Demetrios Kouretas

**Affiliations:** 1 Department of Biochemistry and Biotechnology, University of Thessaly, Viopolis, Mezourlo, 41500 Larissa, Greece; 2 Laboratory of Animal Husbandry, School of Veterinary Medicine, Faculty of Health Sciences, Aristotle University of Thessaloniki, 54124 Thessaloniki, Greece; 3 Faculty of Veterinary Science, University of Thessaly, 43131 Karditsa, Greece

**Keywords:** oxidative stress, antioxidants, redox biomarkers, sheep, goat, meat quality, welfare

## Abstract

The objective of this study was to assess the resting values of the physiological oxidative stress exhibited by lambs and kids reared in Greece, and the potential correlations between redox biomarker levels in blood and other tissues (liver, diaphragm, quadriceps, psoas major muscle). For this purpose, lambs and kids at different developmental stages (d.s.) were used. The latter corresponded to four live weight categories (LWC), each representing 25%, 35%, 70% and 100% of mature body weight. In each of the above tissues, the levels of five common redox biomarkers were determined: glutathione (GSH), catalase (CAT), total antioxidant capacity (TAC), thiobarbituric reactive substances (TBARS), and protein carbonyls (CARBS). The results revealed that lambs and kids belonging to the 35% LWC had weaker endogenous antioxidant pools, while animals in the 70% and 100% LWC had elevated intrinsic antioxidant defense systems. Blood redox biomarkers were associated with the respective ones measured in the diaphragm, liver, quadriceps, and psoas major of both species. Importantly, TBARS levels in blood of animals in the 25% and 100% LWC are correlated with the TBARS levels in all other tissues tested. Blood antioxidant parameters might be used as potential biomarkers to predict the antioxidant status of tissues that affect meat quality. The latter would facilitate quality assessment prior to slaughter, allowing for timely nutritional interventions that can improve meat products.

## 1. Introduction

Global meat production has tripled over the last 50 years in order to address the constantly escalating demand for meat [1]; by 2050, an approximate 73% increase in meat consumption is expected according to the Food and Agricultural Organization of the United Nations (FAO) [2]. The social and economic impact of the expected growth in lamb and kid meat production and consumption, especially in developing countries, may, in the long term, present both positive and negative aspects. The need for more meat is usually related to higher standards of living and it may lead to intensification of production and husbandry practices; however, the implementation of the latter requires careful planning and constant monitoring to avoid negative impacts on animal health, welfare, and product quality. Taking into consideration that ruminant meat appears to be the richest source of conjugated linoleic acid (CLA) [3], a fatty acid with potential health benefits [4], further investigation of ruminant meat properties is gaining additional interest. Therefore, it is critical to determine the levels of endogenous antioxidant content in several tissues of lambs and kids in order to set a solid scientific base in which future studies will follow to describe the impact of the interventions concerning meat product quality and possible unfavorable impacts on human health [5].

Increasing ruminants’ growth rate represents a physiologically demanding process, indulging higher productivity, and might expose their tissues and cells to redox imbalance [6]. This may ultimately impair physiological functions and reduce survival probability and fitness [6]. In response to that, several studies have been conducted in order to depict the hypothesized benefits of antioxidants supplementation in specific periods of ruminants’ productive life [7,8,9]; however, the insights were inconclusive. Specifically, it has been proposed that immoderate feed supplementation and treatment with antioxidants might have a negative impact on animal welfare due to their action as pro-oxidants [10,11]. The presence of metal ions and the concentration of the antioxidant in each environment are crucial factors that might transform an antioxidant into a pro-oxidant [12,13]. Therefore, dispensation of antioxidants individually and only when scientifically proven necessary may constitute a solution under the light of these conflicting results in the literature. In line with the above, a need of reproducible and trustworthy biomarkers that reflect the endogenous antioxidant status of small ruminants using non-invasive techniques is of paramount importance since it will allow reproducibility and comparability of future studies in research or clinical level.

Moreover, weaning and different developmental stages (d.s.) are also associated with changes in animals’ physiological processes. Weaning presents a challenging period for animals, since they are forced to adapt to changes in feed intake, diet form and composition, nutrient digestibility, and social and physical environment [14]. All the above together induce stress [15]. The neonatal period is also very stressful for ruminants [16], since newborns’ adaptation to the extra-uterine life is challenging in terms of physiology and metabolism; young ruminants experience increased oxidative stress during their first exposure to an oxygen-rich environment. Despite the significant impact of changes in the redox status after birth and during the maturation of ruminants on animal health and welfare and consequently meat quality, relevant studies are scarce.

As mentioned before, animal health is affected by oxidative modifications, which can directly modulate oxidation of important macromolecules such as lipids and proteins, leading towards dysregulation of metabolic pathways [5]. To counteract the above, cells encompass safeguard mechanisms to dodge adverse effects of harmful reactive species. These mechanisms include enzymatic and non-enzymatic antioxidants that can also work in synergy [17]. Additionally, taking into account that the oxidative stability of meat depends on the balance between anti- and pro-oxidant substrates [18], the redox status of ruminants could be an important indicator towards meat quality and ruminants’ overall health.

Therefore, it is crucial to determine specific biomarkers on animals that will reflect quality deterioration of their meat products [19]. Substantial also remains the determination of antioxidant parameters such as glutathione, total antioxidant capacity (TAC), and catalase, since they protect meat against quality deterioration during storage [20], permit the capacity of certain molecules included in meat to eliminate free radicals [21], and protect cells from oxidative damage in vivo [22], respectively.

Moreover, the assessment of antioxidant biomarkers basal levels is a prerequisite for suggesting specific interventions at certain d.s. of ruminants [23]. To that end, the aims of this study are; (i) to determine the baseline levels of five widely used redox biomarkers (reduced form of glutathione, catalase, TAC, TBARS, and protein carbonylation) in five distinct tissues (blood, liver, diaphragm, psoas major and quadriceps muscles) of lambs and kids reared in Greece and slaughtered at four distinct d.s., corresponding to four different live weight categories (LWC—representing 25%, 35%, 70% and 100% of mature body weight), and (ii) to search for possible correlations between biomarker levels measured in the liver, the diaphragm, the quadriceps and the psoas major muscle with their respective levels in the blood. All that will allow us to report alterations that are related to tissue and d.s. and will possibly characterize the oxidative condition of ruminants’ tissues without the use of invasive techniques.

## 2. Materials and Methods

### 2.1. Animals

A total of 144 dairy small ruminants (48 kids and 96 lambs) reared in Greece were used for this study. Animals were slaughtered at four d.s., corresponding to four different LWC, representing 25%, 35%, 70% and 100% of mature body weight. For each species, the LWC ranges were calculated based on the mean live weight of adult animals of both sexes of the local populations from the region of Macedonia in Greece. For lambs, the live weight ranges included in each LWC were respectively, less than 17.6 kg, 17.6–27.5 kg, 39.6–55 kg and more than 55 kg. Respective ranges for kids were less than 17.1 kg, 17.1–23.5 kg, 32.6–42.5 kg and more than 42.5 kg. The first two LWC, 25% and 35%, corresponded respectively to weaning and post-weaning d.s., whereas the latter two (70% and 100% LWC) corresponded to young adult and mature animals. Animals belonged to both sexes in equal numbers. The breed, grazing and diet composition of the animals do not concern the present study. No feed additives or antioxidants were used in this study. The slaughtering took place in two approved commercial slaughterhouses, according to EU standards (854/2004). The distance from the animals’ farm to the slaughterhouse was no more than 20 km.

### 2.2. Sample Collection

The procedure was previously described in the connected publication concerning cattle samples [23]. Specifically, blood collection tubes (EDTA, BD Vacutainer^®^ Blood collection tubes, BD, USA) were used for the blood samples collection from the jugular vein. The collection took place during the bleeding of the animals. Immediately after slaughter, tissue collection was performed. Samples of the liver and muscle (i.e., psoas major, quadriceps and diaphragm) were excised (10 g per sample) and kept in plastic cassettes. Briefly, the diaphragm muscle was derived from the pars costalis, due to its easy and rapid accessibility. Concerning the quadriceps muscle, the rectus femoris muscle was sampled, especially the edge near the end of the femur. The psoas major was collected from the muscle part that, together with the gluteus muscle, inserts on the trochanter minor. The part of the liver in which the duodenum is impressed was also collected. Following collection, the samples were immediately placed in liquid nitrogen and transferred within 3 h to be stored at −80 °C until analysis.

### 2.3. Blood Fractionation

The blood samples were centrifuged immediately after collection at 1370× *g* for 10 min at 4 °C. Thereafter, the collected plasma was used to measure the TAC, TBARS and CARBS levels. The packed erythrocytes were lysed with distilled water (1:1 *v*/*v*), inverted vigorously, centrifuged at 4000× *g* for 15 min at 4 °C, and the erythrocyte lysate (RBCL) was collected for the measurement of the GSH and CAT activity. Before the measurement of GSH concentration, 400 μL erythrocyte lysate and 400 μL 5% trichloroacetic acid (TCA) were mixed, inverted vigorously, centrifuged at 15,000× *g* for 5 min at 4 °C, and the supernatant was collected. Then, 90 μL 5% TCA was collected and added to each tube, inverted again vigorously and centrifuged as previously. Finally, the clear supernatant was collected and used for the estimation of GSH concentration.

The hemiglobincyanide (HiCN) method using a commercial kit (Dutch Diagnostics, Zutphen, Holland) was used for the determination of hemoglobin concentrations. Briefly, 5 μL RBCL and 1 mL working hemoglobin reagent (reagent R1) were mixed. The composition of reagent R1 (pH 7.3) is potassium hexacyano ferrate (III) (0.607 mmol/L), potassium cyanide (0.767 mmol/L), potassium dihydrogen phosphate (1.03 mmol/L) and 0.05% detergent. Afterwards the samples were vortexed and incubated in a dark place for 10 min. Each experiment used 1 mL R1 as a blank. Finally, the optical density was measured at 540 nm.

### 2.4. Tissue Homogenization and the Measurement of the Total Protein Concentration

A Bertin Technologies homogenizer was used for tissues homogenization. In brief, 200–250 mg of each sample and 600–750 μL Phosphate Buffer Saline (PBS) with diluted protease inhibitors (CompleteTM mini protease inhibitors) were transferred into homogenization tubes. The samples were homogenized for 30 s at the highest available speed. Then, the homogenate was centrifuged (15,000× *g*, 5 min, 4 °C) and the supernatant crude protein extract was collected in new Eppendorf tubes. The total protein concentration was determined using the Bradford method and the samples were stored at −80 °C until the analysis.

### 2.5. Redox Biomarkers Determination

Each assay was performed in triplicate and within 3 months of the blood collection. The blood samples were aliquoted at −80 °C and thawed once prior to analysis.

#### 2.5.1. Reduced Form of Glutathione (GSH)

The determination of reduced form of GSH was determined by the method of Reddy et al. [24]. Minor modifications of the protocol were previously described by Veskoukis et al. [25] and were included in the present experimental protocol. Briefly, 20 μL erythrocyte lysate or 100 μL tissue homogenate was treated with 5% trichloroacetic acid (TCA) and the mixture was centrifuged (15,000× *g*, 5 min, 5 °C). The supernatant was transferred to a new Eppendorf tube and 20 μL of it was mixed with 660 μL sodium potassium phosphate buffer (67 mM, pH 8) and 330 μL 5,5′-dithiobis-2 nitrobenzoate (DTNB; 1 mM). The reaction solution was incubated in the dark at room temperature for 15 min and the absorbance was read at 412 nm (Hitachi, U-1500).

#### 2.5.2. Catalase (CAT)

The CAT activity was assessed using a slightly modified version of the assay of Aebi [26], as described in the study by Veskoukis et al. [25]. In brief, 4 μL RBCL (dilution of 1:10) or 5 μL liver homogenate (dilution of 1:5) οr diaphragm, psoas major and quadriceps, undiluted, was mixed with 2991 or 2990 μL of 67 mM sodium potassium phosphate buffer (pH 7.4), respectively, followed by incubation at 37 °C for 10 min. After that, 5 μL 30% H_2_O_2_ were added to the mixture and the rate of decrease in optical density was measured for 130 s at 240 nm (Hitachi, U-1500). The calculation of CAT activity was based on the molar extinction coefficient of H_2_O_2_ (43.6 M^−1^ cm^−1^).

#### 2.5.3. Total Antioxidant Activity (TAC)

TAC levels were evaluated according to the method of Janaszewska and Bartosz [27]. The reaction solution was incubated in the dark at room temperature for 60 min in a final volume of 1 mL consisted of 40 μL tissue homogenate or 20 μL plasma, 460 or 480 μL phosphate buffer (10 mM; pH 7.4), respectively, and 500 μL 2,2-diphenyl-1-picrylhydrazyl radical (DPPH, 0.1 mM) solution. The mixture was centrifuged (15,000× *g*, 3 min) and the absorbance was read at 520 nm (Hitachi, U-1500). TAC is presented as mmol of DPPH reduced to 2,2-diphenyl-1-picrylhydrazine (DPPH:H) by the antioxidants of plasma.

#### 2.5.4. Thiobarbituric Acid Reactive Substances (TBARS)

Concerning the assay for TBARS determination, a slightly modified version of the method of Keles et al. [28] was used, as previously described by Spanidis et al. [29]. More specifically, 100 μL plasma or tissue homogenate and 500 μL 35% TCA and 500 μL Tris–HCl (pH 7.4) were mixed. Incubation of the samples for 10 min at room temperature followed. Afterwards, 1 mL containing Na_2_SO_4_ (2M) and thiobarbituric acid (TBA; 55 mM) was added to the mixture. Following 45-min incubation at 95 °C, the samples were transferred at 4 °C for 5 min and vortexed after adding 1 mL of 70% TCA. From each sample, 1 mL was transferred to Eppendorf tubes and the samples were centrifuged (11,200× *g*, 3 min). Finally, 900 μL of the supernatant was transferred into a plastic cuvette and the optical density of the MDA(TBA)2 adduct was determined at 530 nm (Hitachi, U-1500). The calculation of the TBARS concentration was performed using the molar extinction coefficient of malondialdehyde (155 × 103 M^−1^ cm^−1^).

#### 2.5.5. Protein Carbonyls

A slightly modified version of the method of Patsoukis et al. [30] was used for the determination of protein carbonyl content, as previously described by Veskoukis et al. [25]. Briefly, 50 μL 20% TCA and 50 μL plasma or tissue homogenate were mixed. The mixture was incubated for 15 min at 4 °C, followed by centrifugation (15,000× *g*, 5 min, 4 °C). Subsequently, the supernatant was discarded, and the pellet was resuspended in 500 μL 10 mM 2,4-dinitrophenylhydrazine (DNPH) (diluted in 2.5 N HCl). Every sample had its own blank that constituted of the same sample volume resuspended with 500 μL 2.5 N HCl without DNPH. Following resuspension, the samples and blanks were incubated in the dark at room temperature for 1 h with intermittent vortexing every 15 min, followed by centrifugation (15,000× *g*, 5 min, 4 °C). After the centrifugation, the supernatant was removed, and the pellets were resuspended with 1 mL TCA (10%). Following resuspension, the samples and blanks were again centrifuged (15,000× *g*, 5 min, 4 °C) and the pellets were cleaned with 3 washes with 1 mL ethanol–ethyl acetate mixture (1:1 *v*/*v*). The pellets were resuspended while the samples and blanks were centrifuged each time at 15,000× *g* for 5 min at 4 °C. After the third wash, the pellets were resuspended with 1 mL urea (5 M; pH 2.3) and the samples or blanks were vortexed and incubated at 37 °C for 15 min. The samples and blanks were centrifuged (15,000× *g*, 5 min, 4 °C) and their optical density was determined at 375 nm (Hitachi, U-1500). The calculation of the protein carbonyl concentration was based on the molar extinction coefficient of DNPH (22 × 103 M^−1^ cm^−1^).

### 2.6. Statistical Analyses

Data were analyzed by one-way ANOVA followed by post hoc tests for multiple comparisons between the different d.s.; between groups, differences were analyzed separately for each tissue. In the case that normal distribution was not found with Shapiro-Wilk test, we continued using the Kruskal–Wallis H test followed by post hoc tests for multiple comparisons between the different d.s. The level of statistical significance was set at *p* < 0.05. All results are expressed as mean ± standard error of the mean (SEM). The correlation analysis was performed, and the respective Pearson r co-efficiencies were determined to assess possible correlations between the tested parameters in the blood and the other tissues. The statistical analyses were performed using GraphPad Prism 8.0.0.

## 3. Results

### 3.1. Redox Biomarkers Evaluated in 4 Developmental Stages

#### 3.1.1. CARBS

**Figure 1:**
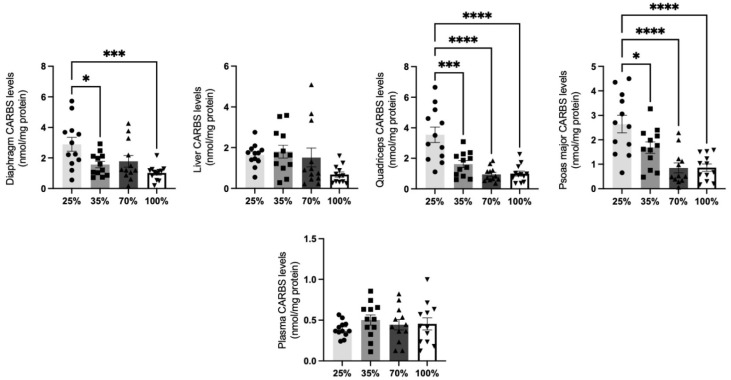
CARBS levels at all 4 d.s. of kids. * denotes significant difference with *p* < 0.05, *** denotes significant difference with *p* < 0.001, **** denotes significant difference with *p* < 0.0001.

Protein carbonylation is a non-reversable post-translational modification that forms as a result of denaturation or proteolysis, and plays a decisive role in the nutritional and quality traits of meat [31]. Additionally, excessive protein carbonylation is toxic and can impair cellular viability [32], since oxidatively modified proteins can form large aggregates [33]. This process allowed us to determine to what extent cellular oxidative stress affected the protein content in the tissues tested, in all four different d.s. Regarding kids (Figure 1), significantly (*p* < 0.05) elevated levels of protein oxidation were found in the psoas major and the quadriceps at the 25% d.s. (weaning) compared to other d.s. (Figure 1). Diaphragm revealed the same pattern, however reduction in CARBS levels was statistically significant only at the 35% and 100% d.s. (*p* < 0.05). In the liver, a trend (*p* < 0.1) for reduction at the 100% d.s. compared to the 25% d.s. was observed. To our surprise, even though CARBS were diminished in the liver and the muscle tissues tested, the respective levels in the plasma remained stable among different d.s. (Figure 1).

**Figure 2:**
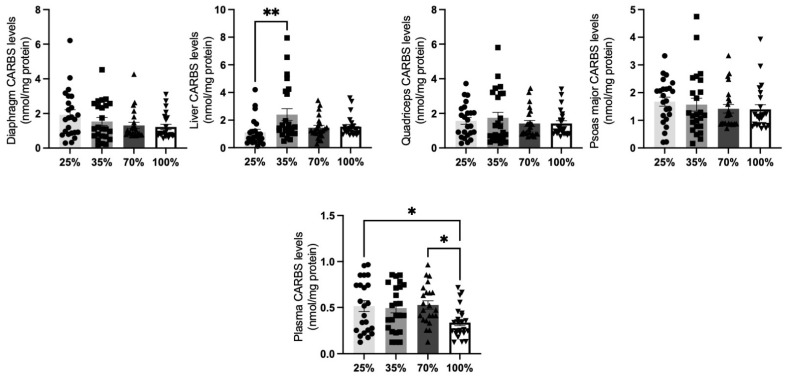
**CARBS levels at all 4 d.s. of lambs**. * denotes significant difference with *p* < 0.05, ** denotes significant difference with *p* < 0.01.

CARBS levels were reduced only in the blood of the lambs that were at the 100% d.s. (*p* < 0.05, Figure 2). Additionally, after an initial increase at 35% d.s., CARBS at the liver of lambs at 70% and 100% d.s. returned to the levels that were observed at the 25% d.s. No other significant change was observed in all other tissues tested.

#### 3.1.2. Catalase Activity

**Figure 3:**
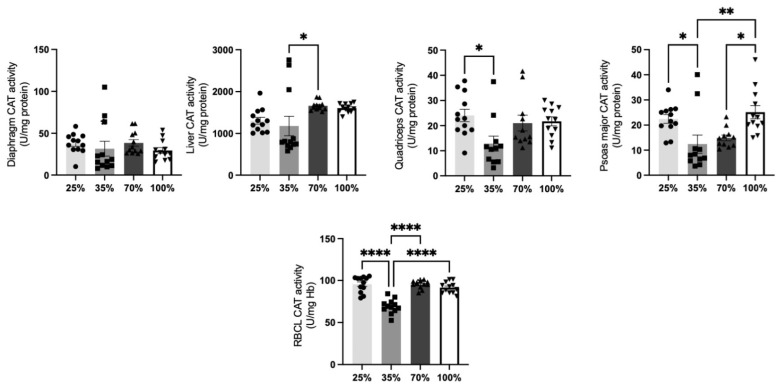
CAT activity levels at all 4 d.s. of kids. * denotes significant difference with *p* < 0.05, ** denotes significant difference with *p* < 0.01. **** denotes significant difference with *p* < 0.0001

Since catalase is one of the most abundant enzymes in the cell with antioxidant properties, CAT activity determination can reflect the antioxidant state of the tissue tested [34]. CAT activity (Figure 3) at the quadriceps, the psoas major, and the RBCL was significantly (*p* < 0.05) reduced at the 35% compared to the 25% d.s. of the kids. Nevertheless, CAT activity at the 70% and 100% d.s. returned to weaning levels of 25 d.s. (Figure 3). CAT activity was elevated in the at the 70% d.s. compared to the 35% d.s. No other significant differences were revealed (*p* > 0.05) (Figure 3).

**Figure 4:**
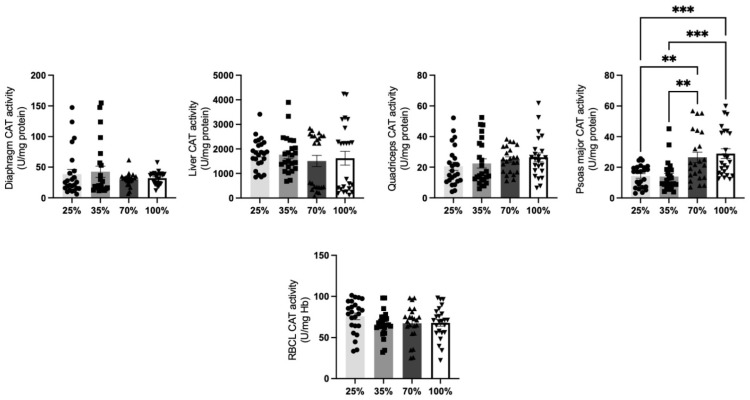
CAT activity levels at all 4 d.s of lamb.** denotes significant difference with *p* < 0.01, *** denotes significant difference with *p* < 0.001.

Concerning CAT activity levels in lambs (Figure 4), a significant (*p* < 0.05) increase in the psoas major was found at 70% and 100% d.s. compared to weaning (25%) and post-weaning d.s. (35%). No other significant differences in CAT activity of lambs’ tissues among the four different d.s. tested (Figure 4).

#### 3.1.3. Glutathione Levels

**Figure 5:**
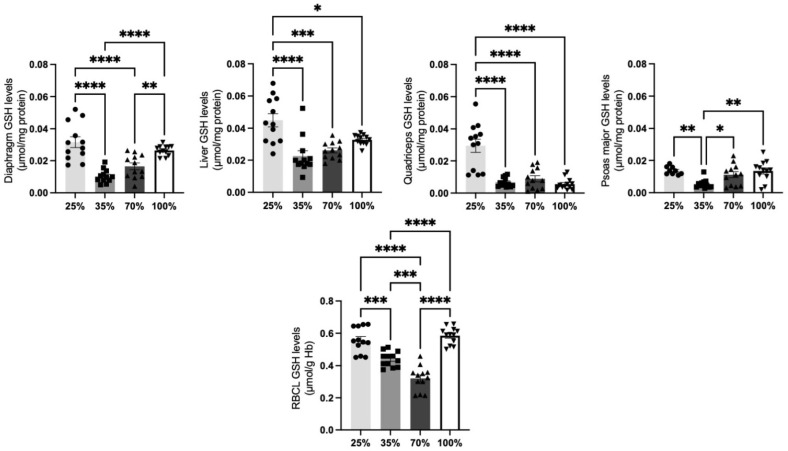
GSH levels at all 4 d.s. of kids. * denotes significant difference with *p* < 0.05, ** denotes significant difference with *p* < 0.01, *** denotes significant difference with *p* < 0.001, **** denotes significant difference with *p* < 0.0001.

GSH, the most abundant intracellular, non-enzymatic, small thiol molecule, is an important antioxidant, playing a critical role in the detoxification of a variety of electrophilic compounds and peroxides. The depletion of GSH levels drives the cell towards oxidative damage, resulting in the onset or the consequence of a wide range of pathologies [35]. All kids’ tissues tested (Figure 5) had a significantly (*p* < 0.05) decreased glutathione content at the 35% and 70% d.s. compared to the 25% d.s., except for the psoas major, in which the decrease was detected only at the 35% d.s. However, GSH content in the diaphragm, the psoas major and the RBCL at the 100% d.s. returned to the levels detected at the 25% d.s. On the contrary, GSH levels at the liver and the quadriceps tissues diminished at all d.s. stages after the weaning period (25% d.s) indicating that these tissues do not require enhanced pool of GSH levels during this developmental period of life, possibly due to less stressful conditions during the 25% d.s. (Figure 5).

**Figure 6:**
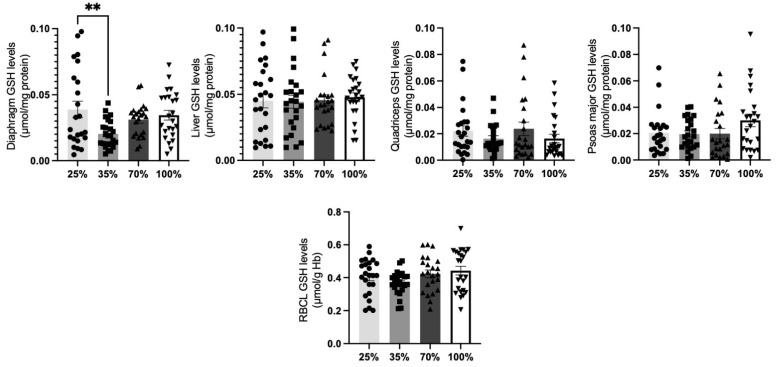
GSH levels at all 4 d.s. of lambs. ** denotes significant difference with *p* < 0.01.

GSH levels in lamb tissues did not proclaim significant alterations across the examined d.s. (*p* > 0.05), apart from a significant (*p* < 0.05) decrease in the diaphragm at 35% compared to the 25% d.s. (Figure 6).

#### 3.1.4. Total Antioxidant Capacity

**Figure 7:**
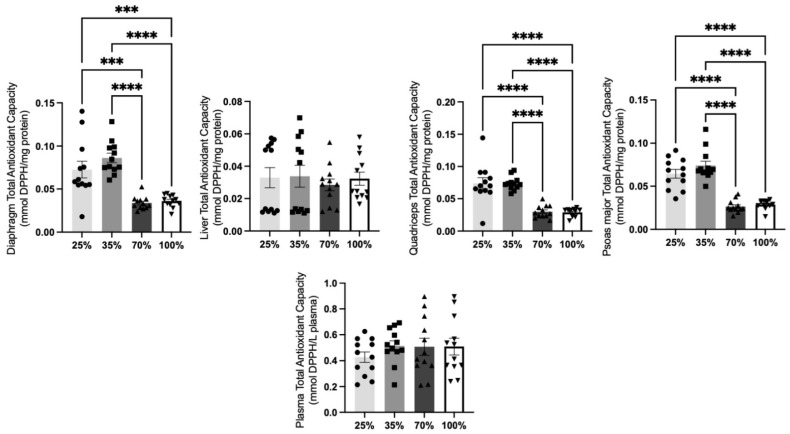
TAC levels at all 4 d.s. of kids. *** denotes significant difference with *p* < 0.001, **** denotes significant difference with *p* < 0.0001.

A diminished antioxidant defense system can lead to cellular damage and dysfunctional tissues. To prevent pathologies, it is essential to maintain the total antioxidant levels (TAC) that represent molecules such as uric acid [36]. TAC levels were significantly (*p* < 0.05) decreased in all tested tissues of kids apart from plasma when compared to 25% and 35% d.s.(Figure 7).

**Figure 8:**
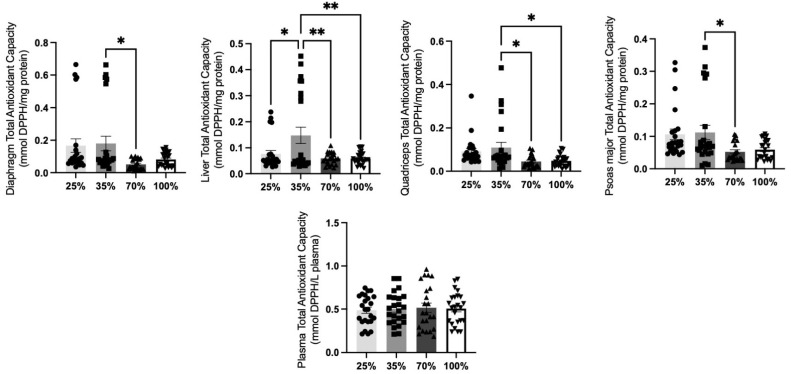
TAC levels at all 4 d.s. of lambs. * denotes significant difference with *p* < 0.05, ** denotes significant difference with *p* < 0.01.

Regarding TAC levels in the lambs, a significant (*p* < 0.05) decrease was observed at 70% d.s. compared to 35% d.s. in all tissues tested, except for plasma, showcasing a pattern of antioxidant decadency after post-weaning period (Figure 8). Liver and quadriceps tissues from lambs at 100% d.s. revealed significantly decreased TAC levels (*p* < 0.05) compared to 35% d.s. However, higher liver TAC levels were reported at 35% d.s. compared to 25% d.s. (Figure 8). Plasma TAC levels did not show any significant (*p* > 0.05) differences among all four d.s. tested (Figure 8).

#### 3.1.5. Thiobarbituric Acid Reactive Substances (TBARS)

**Figure 9:**
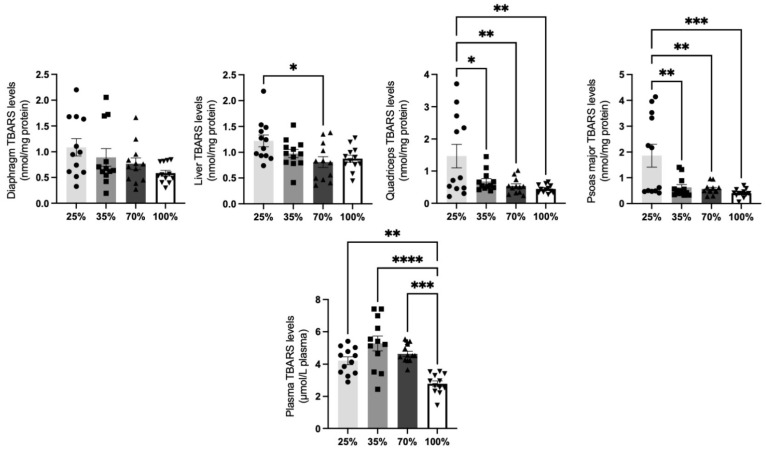
TBARS levels at all 4 d.s. of kids. * denotes significant difference with *p* < 0.05, ** denotes significant difference with *p* < 0.01, *** denotes significant difference with *p* < 0.001, **** denotes significant difference with *p* < 0.0001.

Lipid peroxidation represents a crucial factor for nutritional loss and degradation, and is an important component of measurements for the meat industry. A significant (*p* < 0.05) decrease in TBARS levels in the quadriceps and the psoas major muscle was found in all studied d.s. of kids post weaning (25% d.s.) and also in the liver at 70% d.s. compared to 25% d.s. (Figure 9). TBARS levels in the diaphragm and liver revealed only slighter changes as observed by a trend of decrease in 100% d.s. compared to 25% d.s. (*p* < 0.1) (Figure 9). Plasma lipid peroxidation of kids at the35% d.s. was found to be slightly increased (*p* < 0.1) after the weaning period (25% d.s.) but afterwards revealed a significant decrease at 100% d.s. compared to 25% and 35% d.s., denoting the same pattern that has been described in the other tested tissues (*p* < 0.05) (Figure 9).

**Figure 10:**
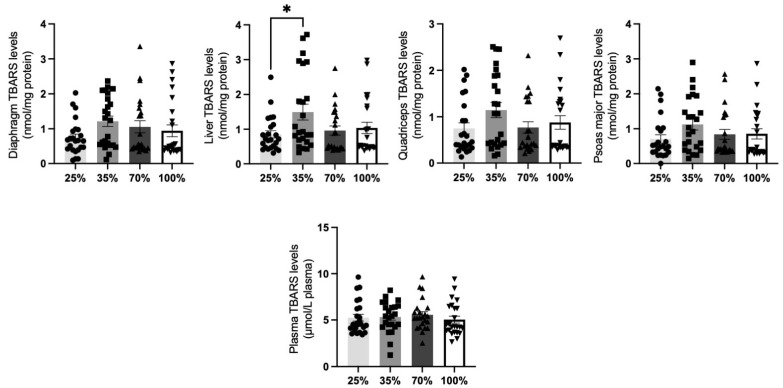
TBARS levels at all 4 d.s of lambs. * denotes significant difference with *p* < 0.05.

In lambs, TBARS levels of all tissues remained stable among d.s. (*p* > 0.05). The only exception was detected in the liver, in which a significant (*p* < 0.05) increase was found at 35% d.s. compared to 25% d.s. (Figure 10).

### 3.2. Correlation Analysis

Significant correlations between redox biomarkers in blood and the other four tissues tested in kids and lambs are presented in Table 1 and Table 2, respectively. Concerning kids, diaphragm CAT activity in 25% d.s., psoas major CARBS levels in 70% d.s. and diaphragm GSH levels in 100% d.s. were significantly positively correlated with the respective biomarkers in blood samples (Table 1). Furthermore, TBARS in the diaphragm, liver, quadriceps and the psoas major from 25% d.s. kids were positively correlated with TBARS in the blood (Table 1). The same pattern was also observed in the TBARS from all tissue tested from 100 d.s. kids, except for the diaphragm, which revealed only a slight trend towards a positive correlation. On the other hand, diaphragm TAC levels from 25% d.s. kids were negatively correlated with their respective TAC levels in the blood (Table 1). The rest of the linear regression analyses did not reveal any significant correlations between biomarkers measured in the blood and the other four tissues tested.

Concerning lambs, CARBS in the liver at 25% d.s., TBARS in the psoas major at 35% d.s., GSH in the diaphragm at 70% d.s., CAT in the liver at 100% d.s., were positively correlated with the respective biomarkers in the blood. Similarly, TBARS in the diaphragm, liver and quadriceps at 100% d.s. were positively correlated with TBARS in the plasma from the same developmental stage. On the contrary, negative correlations were detected between CAT in the diaphragm at the 35% d.s., GSH in the quadriceps at the 35% d.s., the GSH in psoas major at the 100% d.s., and the respective biomarkers in blood from the same animals (Table 2). The rest of the linear regression analyses did not reveal any significant correlations between biomarkers measured in the blood and the other four tissues tested. 

## 4. Discussion

To our knowledge, the present study is the first to investigate the baseline levels of various redox biomarkers in lambs and kids reared in Greece that were slaughtered at four different developmental stages (d.s.; 25%, 35%, 70% and 100%). Moreover, this study aimed to assess the association of biomarkers tested in the blood with the other tissues in order to allow animal health screening and possible nutritional interventions without invasive techniques. It constitutes a follow-up study representing a holistic project [23], that investigates the oxidative stability and quality of lambs, kids, and cattle meat in a large scale of animal samples from different d.s. The chosen approach manifests its dominance towards previous small-scale studies, because of the selection of the same anatomical sites in the tissue sampling and the selection of animals that were not receiving additional nutritional interventions, enabling us to compare redox baseline levels of commonly consumed meat in Greece. The results revealed that, in goats post-weaning (35% d.s.), antioxidant status was more diminished as manifested by lower GSH, CAT and TAC levels, whilst the oxidation of proteins and lipids was also reduced. The examined biomarker levels in lambs did not reveal a solid pattern, since both antioxidant and oxidative biomarkers were evenly enhanced or diminished, following possibly mutual adaptations between developmental stages. Moreover, a strong correlation was reported between TBARS levels measured in the blood and the rest of the tested tissues, suggesting the possibility of avoiding invasive methodologies in future research or clinical setups.

Our results suggest that kids have a distinct profile in the antioxidant status as determined by GSH, CAT, and TAC after weaning. Specifically, GSH levels in all tissues examined revealed an acute decrease after the 25% d.s., showing an optimal stress condition in these animals. It has been previously reported that weaning stress is a crucial factor that might negatively affect the feed intake and antioxidant status of the animals [37]. Specifically, kids exert higher malondialdehyde (MDA) levels and lower superoxide dismutase (SOD) activity in their lower gut after weaning [37]. Additionally, other studies describe the nutritional, social, and environmental changes that affect weaning conditions, demonstrating that it is likely to be a health-challenging period [38,39]. Pigs demonstrated decreased plasma SOD activity in combination with increased concentrations of MDA, NO and H_2_O_2_ after weaning [40]. Furthermore, weaning has resulted in a decrease in the expression of several genes encoding antioxidant enzymes and the levels of tumor protein 53, stimulating ROS generation [40]. The levels of PGC-1a, a master regulator of mitochondrial function and mitochondrial detoxification proteins, have also been reduced in weanling pigs compared to control animals [40]. In the present study, CAT activity was also found to be downregulated in quadriceps, psoas major and RBCL at 35% d.s of the kids., but in later d.s. the depletion seems to be restored. Available data show that a high percentage of farmers supplement their animals with additives of vitamins and trace elements post-weaning as a precaution against diseases as well as for improving animal performance and productivity [41]. Regarding TAC levels in kids’ tissues, our study found that were mainly affected during the 70% and 100% d.s., in all tissues tested except for plasma. On the contrary, the rest of the antioxidant biomarkers (GSH levels and CAT activity) tested were affected at earlier developmental stages (25% and 35%). TBARS levels in kids were mostly affected during weaning and post-weaning period in the plasma, the quadriceps, and the psoas major muscle. With the exception of increased plasma lipid peroxidation at weaning (25% d.s.), the rest of the tissues showed gradually decreased levels in 35%, 70% and 100% d.s., suggesting their ability to counteract the oxidative stress induced in this crucial time period. In consonance with this decrease in lipid peroxidation, antioxidant molecules like GSH were consumed at these d.s. Moreover, another study revealed the induction of hepatic oxidative stress as indicated by high TBARS levels in post-weaning piglets, basically related to the activated Mitogen-Activated Protein Kinases (MAPK) pathways [42]. In the present study, protein oxidation levels in kids follow the same pattern as TBARS in quadriceps and psoas major muscles, indicating a proposed counteraction of the weaning generated free radicals. Moreover, a study that tried to shed light into oxidative stress and the development of an antioxidant system after early weaning in piglets has shown that plasma protein carbonyl levels are increased from day 1 to day 5 after early weaning [43]. The authors suggest that even though weaning can alter the oxidant/antioxidant balance in piglets, this balance seems to be restored with the boost in components of the antioxidant system via feedback regulation [43].

The results pertaining to lamb redox biomarkers levels and activity revealed minimum changes from birth to post-weaning and maturation stages. The clearest and most prominent results are related to TAC and CARBS levels. TAC levels in the diaphragm, liver, quadriceps, and psoas major muscle were decreased in weaned lambs following the same pattern as kids. Plasma and liver CARBS levels were significantly decreased in the post-weaning period, with a slight increase in the weaning period in liver tissue, showcasing the capability of the endogenous antioxidant system to counteract the oxidation of critical biomolecules post-natal and after the weaning period. Altogether, the results in post-weaned lambs and kids indicate the restoration of their antioxidant repository, which may be a specific type of pattern throughout their lifetime. Based on these, recommendations for nutritional interventions only in weaned ruminants, and when their redox status seems to be deregulated, could compose an important improvement for their performance in this specific d.s. The combined redox status investigation and intervention whenever needed could confer substantial individual health benefits in ruminants, improve competitive ability, enhance thermoregulation, and increase survival probability and productivity.

The linear regression analyses between blood and liver or different muscle antioxidant/oxidant status biomarkers were evaluated in order to find prognostic blood biomarkers for the estimation of oxidative stability of tissues and consequently meat products that derive from different d.s. This is the first attempt to examine this type of correlation coefficient in kids and lambs from Greece in a large-scale study. While a previous study indicated that plasma GSH levels have been highly correlated with the intrahepatic level [44], limited data exist on whether multiple redox biomarkers in small ruminants’ blood are correlated with liver, diaphragm, quadriceps, and psoas major muscle. The present results showed high correlation coefficients for TBARS levels between blood and the rest of the tissues tested in kids and lambs. Specifically, according to the present study, the desirable decrease in TBARS levels in meat from kids in 25% and 100% d.s. could be predicted by a simple blood measurement. Concerning TBARS levels, no significant correlations in redox status indices between the blood and skeletal muscles were found previously [45]; nevertheless, our results in lambs reported a positive correlation in the psoas major of 35% d.s. animals as well as with the diaphragm, liver, and quadriceps belonging to 100% d.s. animals. Another study has provided evidence that TBARS measured in the blood sufficiently describe the redox status of the heart and liver of rats [46]. Protein carbonylation in the plasma tested in our study showed minimal correlation with the psoas major muscle and liver at the 70% and 25% d.s. of lambs, respectively. Our results relating to correlation coefficients of antioxidant status biomarkers were not so pronounced. In most cases, the diaphragm revealed a positive correlation with blood antioxidant levels as determined by all the tested methods in lambs and kids. The quadriceps, psoas major, and liver revealed only a few correlation coefficients with the blood of lambs. In line with the above, protein carbonyls, GSH, and catalase activity measured in the blood can adequately describe the skeletal muscle and heart redox status [46].

## 5. Conclusions

The present results indicate a decrease in antioxidant status post weaning (35% d.s.) in lambs and kids. This impact can subsequently be counteracted by the endogenous system of the animals, since GSH, CAT and TAC levels in specific tissues of lambs and kids jumped back as seen at 70% and 100% d.s. Moreover, based on the present findings, it seems that in specific d.s., oxidative stress markers alter in a particular direction in the blood and simultaneously in the quadriceps, liver, diaphragm, and the psoas major. This implies that changes detected in blood biomarkers may also reflect alterations in the redox status of several tissues. Noteworthily, TBARS changes detected in the blood may also reflect alterations in lipid peroxidation levels that were exhibited mainly in the goat tissues. The assessment of redox status in blood could comprise a useful tool for animal husbandry for evaluating the quality of livestock carcasses and for establishing new strategies and interventions for improved goat meat products. Further research is needed in order to strengthen the applicability of our results in other animal species.

## Figures and Tables

**Table 1:** Linear regression analyses between redox biomarkers in kids’ blood and the other 4 tissues tested (liver, diaphragm, quadriceps, psoas major) at the same d.s. (*p* < 0.05, r > 0.5).

D.s.	Biomarker	Tissues	r	*p*-Value
70%	CARBs	Blood-psoas major	0.6547	0.0209
25%	CAT	Blood-diaphragm	0.6582	0.0200
100%	GSH	Blood-diaphragm	0.6364	0.0261
25%	TAC	Blood-diaphragm	−0.5919	0.0426
25%	TBARS	Blood-diaphragm	0.6583	0.0139
25%	TBARS	Blood-liver	0.6600	0.0195
25%	TBARS	Blood-quadriceps	0.6955	0.0120
25%	TBARS	Blood-psoas major	0.7867	0.0024
100%	TBARS	Blood-liver	0.8226	0.0010
100%	TBARS	Blood-quadriceps	0.6406	0.0248
100%	TBARS	Blood-psoas major	0.5677	0.0442

**Table 2:** Linear regression analysis between redox biomarker levels in lambs’ blood and the other 4 tissues tested (liver, diaphragm, quadriceps, psoas major) at the same d.s. (*p* < 0.05, r > 0.5).

D.s.	Biomarker	Tissues	r	*p*-Value
25%	CARBS	Blood-liver	0.4412	0.0398
35%	CAT	Blood-diaphragm	−0.4118	0.0456
100%	CAT	Blood-liver	0.4276	0.0371
35%	GSH	Blood-quadriceps	−0.4703	0.0204
70%	GSH	Blood-diaphragm	0.4398	0.0357
100%	GSH	Blood-psoas major	−0.5036	0.0121
35%	TBARS	Blood-psoas major	0.4809	0.0174
100%	TBARS	Blood-diaphragm	0.4837	0.0166
100%	TBARS	Blood-liver	0.4034	0.0406
100%	TBARS	Blood-quadriceps	0.6030	0.0018

## Data Availability

The data described in this study are available upon request from the corresponding author.

## Abbreviations

GSH	reduced glutathione
CAT	catalase
TAC	total antioxidant capacity
TBARS	thiobarbituric acid reactive substances
CARBs	protein carbonyls
d.s.	developmental stage
LWC	live weight category
